# Effectiveness of Combined Health Coaching and Self-Monitoring Apps on Weight-Related Outcomes in People With Overweight and Obesity: Systematic Review and Meta-analysis

**DOI:** 10.2196/42432

**Published:** 2023-04-18

**Authors:** Han Shi Jocelyn Chew, Nagadarshini Nicole Rajasegaran, Yip Han Chin, W S Nicholas Chew, Kyung Mi Kim

**Affiliations:** 1 Alice Lee Centre for Nursing Studies Yong Loo Lin School of Medicine National University of Singapore Singapore Singapore; 2 Yong Loo Lin School of Medicine National University of Singapore Singapore Singapore; 3 Department of Cardiology National University Hospital Singapore Singapore Singapore; 4 Office of Research Patient Care Services Stanford Health Care Menlo Park, CA United States

**Keywords:** apps, BMI, cardiometabolic, database, diet behaviour, health coaching, monitoring, obesity, physical activity, waist circumference, weight loss, weight

## Abstract

**Background:**

Self-monitoring smartphone apps and health coaching have both individually been shown to improve weight-related outcomes, but their combined effects remain unclear.

**Objective:**

This study aims to examine the effectiveness of combining self-monitoring apps with health coaching on anthropometric, cardiometabolic, and lifestyle outcomes in people with overweight and obesity.

**Methods:**

Relevant articles published from inception till June 9, 2022, were searched through 8 databases (Embase, CINAHL, PubMed, PsycINFO, Scopus, The Cochrane Library, and Web of Science). Effect sizes were pooled using random-effects models. Behavioral strategies used were coded using the behavior change techniques taxonomy V1.

**Results:**

A total of 14 articles were included, representing 2478 participants with a mean age of 39.1 years and a BMI of 31.8 kg/m2. Using combined intervention significantly improved weight loss by 2.15 kg (95% CI −3.17 kg to −1.12 kg; *P*<.001; I2=60.3%), waist circumference by 2.48 cm (95% CI −3.51 cm to −1.44 cm; *P*<.001; I2=29%), triglyceride by 0.22 mg/dL (95% CI −0.33 mg/dL to 0.11 mg/dL; *P*=.008; I2=0%), glycated hemoglobin by 0.12% (95% CI −0.21 to −0.02; *P*=.03; I2=0%), and total calorie consumption per day by 128.30 kcal (95% CI −182.67 kcal to −73.94 kcal; *P*=.003; I2=0%) kcal, but not BMI, blood pressure, body fat percentage, cholesterol, and physical activity. Combined interventional effectiveness was superior to receiving usual care and apps for waist circumference but only superior to usual care for weight loss.

**Conclusions:**

Combined intervention could improve weight-related outcomes, but more research is needed to examine its added benefits to using an app.

**Trial Registration:**

PROSPERO CRD42022345133; https://tinyurl.com/2zxfdpay

## Introduction

Obesity is an ongoing, serious, and costly health issue at the global level [1] Approximately 2 billion adults were overweight worldwide in 2016, representing approximately 39% of the global adult population [2], and this is projected to affect half of the world’s population by 2030 [3]. The global cost of obesity was estimated at approximately US $2 trillion annually, which is equivalent to the economic burden caused by smoking [3]. Effective interventions for obesity involve multiple components that combine educational, environmental, and behavioral strategies to promote healthy eating and physical activity. Mobile health (mHealth) technologies, such as smartphone apps and wearable devices, have been used widely as promising strategies to enhance the effectiveness of weight loss interventions [4]. Key components of a weight loss app include self-monitoring, tailored behavior change recommendations, and just-in-time reminders [5]. Recent systematic reviews found that the use of mHealth technology was associated with greater weight loss because it allowed for more efficient self-monitoring and analysis of dietary intake, physical activity, and weight [4,6]. Furthermore, the use of technology in conjunction with other strategies, such as health coaching, feedback, and follow-up, could have a greater impact on successful weight loss [4,6]. Health coaching, as with other avenues for weight loss that coincide with mHealth technologies, may provide emotional and knowledge support, thus motivating an individual to modify their lifestyle and adhere to the treatment to a greater degree [6,7].

However, there is a paucity of evidence on the use of mHealth technology in conjunction with health coaching on the outcomes of obesity. Due to the limited use of a health coaching definition in the existing literature on the effectiveness of health coaching interventions for weight loss, we defined health and wellness coaching as a patient-centric process whereby coaches assist clients to use insight, personal strengths and resources, goal setting, action steps, and accountability to achieve a healthy lifestyle change [8]. This is in line with the definition by the National Consortium for Credentialing Health and Wellness Coaches (NCCHWC), which adds that the “health and wellness coaches are professionals from diverse backgrounds and education” [9]. This includes 5 main criteria, namely (1) being partially or fully patient-centered, (2) setting patient-determined goals, (3) including active learning processes about self and health, (4) promoting behavioral accountability, and (5) patient education [10]. Few studies evaluated whether the incorporation of health coaching with mHealth technology is effective to improve clinical outcomes for obesity. In addition, previous studies included wide variations of technologies and health-coaching interventions. Thus, we conducted a systematic review to evaluate the effectiveness of combining weight management apps with health coaching on clinical outcomes among people with overweight and obesity. We specifically focused on smartphone apps because these are the most commonly used for weight loss [4].

## Methods

We conducted this systematic review and meta-analysis according to the PRISMA (Preferred Reporting Items for Systematic Reviews and Meta-Analyses) guidelines and registered it with the International Prospective Register of Systematic Reviews (PROSPERO; ref: CRD42022345133) [11].

### Search Strategy

Seven electronic databases (ie, Embase, CINAHL, PubMed, PsycINFO, Scopus, The Cochrane Library, and Web of Science) were searched for articles examining the effectiveness of smartphone apps on influencing weight loss outcomes from the journals’ inception till June 9, 2022. The search strategy included key search terms and medical subject headings, such as “overweight,” “obesity,” “smartphone apps,” and “health coaching,” and the full search strategy can be found in Table S1 (Multimedia Appendix 1). All citations found were managed using EndNote X20 (Clarivate).

### Study Selection

Two authors (HSJC and NNR) independently screened through the titles and abstracts retrieved. The full text of the shortlisted articles was then examined and articles that fitted the inclusion criteria were marked for inclusion. All discrepancies were resolved through discussions with the reviewers, and where necessary, an independent third senior author (KMK) was consulted. The eligibility criteria for article inclusion were defined based on the population, intervention, comparator, outcomes, and study design framework, and are as follows: (1) randomized controlled trials examining adult participants (aged more than 18 years) with overweight or obesity, (2) weight loss programs that contain a self-monitoring app and health coaching (to fulfill the 5 aforementioned criteria), and (3) examined the primary outcome of weight loss of participants. An article would qualify for the criteria “partially or fully patient-centered” as long as it included active discussion and shared decision-making between the app and patients to provide tailored recommendations. Gray literature (eg, conference abstracts and proceedings), secondary studies (eg, literature review), and observational studies (eg, cross-sectional research studies) were excluded. Studies that examined the participants with mental health disorders, such as major depressive disorder and chronic diseases (eg, heart failure), or pediatric populations were excluded. We also excluded programs that did not have an app component beyond the function of conversation (eg, only SMS text messages through chatting apps, such as WhatsApp, or phone calls). Duplicated studies that obtained results from the same databases were removed, and the latest or the most comprehensive publication was retained.

### Data Extraction

Data were independently extracted by 2 reviewers (NNR and HSJC) using a prepiloted Excel spreadsheet. This includes information regarding the article characteristics (author, country, clinical trial registration number or code, year of study, intervention duration, follow-up time points, theoretical framework, and treatment fidelity), baseline information of included participants (sample size, sample characteristics, mean age, BMI, cutoff point, and mean BMI), intervention characteristics (qualification of interventionist, intervention type [group vs individual, automated vs human guidance]), intervention components (coded by HSJC according to the behavior change techniques taxonomy V1 [12]), app name, external monitoring device, number of coaching sessions, and the study outcomes. All continuous outcomes were extracted in mean (SD or SE) or mean difference (95% CI). Sample variances reported in CIs and SEs were converted to SD, and missing SDs were calculated using *P* values [13]. Weight was collected in kg; waist circumference (WC) in cm; and total cholesterol (TC), low-density lipoprotein cholesterol (LDL-C), high-density lipoprotein cholesterol (HDL-C), and triglyceride were collected in mmol. Glycated hemoglobin (HbA_1c_) was reported as a percentage (%).

### Methodological Quality Assessment

The Cochrane Risk of Bias (ROB) tool was used to assess the methodological quality of the included articles on 7 domains, namely random sequence generation, allocation concealment, participant blinding, evaluator blinding, incomplete outcomes, selective reporting, and other biases [14]. Articles were given a rating of low, unclear, and high ROB independently by 2 authors (NNR and HSJC). Discrepancies were resolved through discussions between the reviewers.

### Data Analysis

All statistical analyses were performed using R version 4.1.3 [15]. A comparative meta-analysis was used to compare primary and secondary outcomes at the first postintervention follow-up. Cardiometabolic outcomes (ie, weight, BMI, WC, blood pressure, TC, LDL-C, HDL-C, triglyceride, and HbA_1c_) and total calorie consumption were estimated using weighted mean differences (WMDs). Body fat percentage and physical activity were estimated using standardized mean differences. Standardized mean differences were expressed as Hedges *g* to account for the small number of studies included in the meta-analyses. Meta-analyses were conducted using random-effects models to account for heterogeneity in interventional effects due to differences, such as sample characteristics and intervention components. Meta-analyses were also adjusted using the Hartung-Knapp-Sidik-Jonkman method rather than the commonly used DerSimonian-Laird method as they account for sampling error and small sample size [16,17]. τ^2^ was used to estimate the between-study heterogeneity, and *I^2^* statistic was used to quantify the heterogeneity where 25%, 50%, and 75% indicate a small, moderate, and large degree of heterogeneity, respectively [18]. Heterogeneity was investigated through subgroup analyses and meta-regressions of potential moderators namely whether the intervention group was compared to a control group with or without the use of an app and the number of health coaching sessions using mixed-effects models [18]. Funnel plots and Egger tests were used to assess for publication bias when there are more than 10 studies included in the meta-analysis [19].

In cases of 3-arm studies, we only used comparisons between combined app and health coaching versus app only, or combined app and health coaching versus usual care (control group without app and health coaching). We did not examine the comparison between health coaching plus app combination versus health coaching only, as it is not the focus of the study. In cases of 3-arm studies that included 2 different app-based weight loss programs, pooled intervention outcome data were extracted. Discrepancies if any were resolved through discussions. Primary outcomes included weight loss, and secondary outcomes included cardiometabolic markers, eating behaviors, and physical activity.

## Results

### Overview

**Figure 1:**
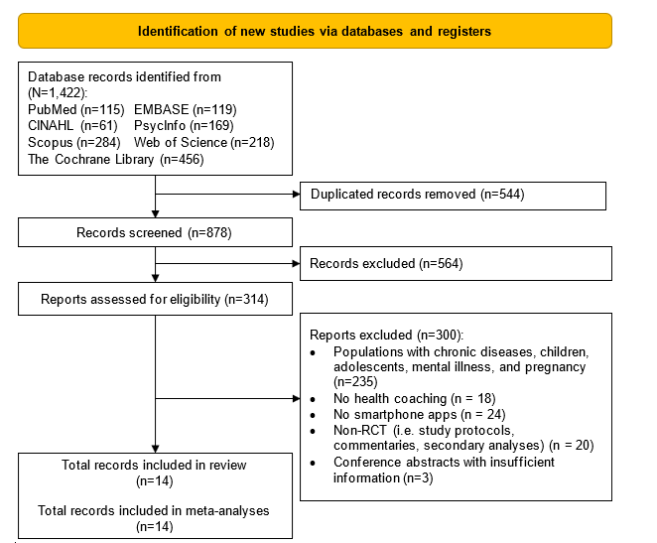
Flow diagram of the search strategy. RCT: randomized controlled trial.

Our database search retrieved 1422 articles, of which 544 were duplicated articles. A total of 314 full texts were assessed for inclusion eligibility of which 300 articles were excluded with reasons (Figure 1), resulting in 14 articles included and 78 unique effect sizes meta-analyzed. The interrater agreement statistics for the inclusion of the article and the overall ROB were κ=0.85 (*P*=.001) and κ=0.74 (*P*<.001), respectively, indicating moderate agreements.

### Study and Intervention Characteristics

Sample characteristics of the 14 included articles are detailed in Table 1. The included studies represent 2478 participants with a mean age of 39.1 years and a mean BMI of 31.8 kg/m^2^. Most of the included articles were from the United States (10/14, 71.4%) [20-29], and one each from Australia (1/14, 7.1%) [30], Belgium (1/14, 7.1%) [31], Korea (1/14, 7.1%) [32], and Japan (1/14, 7.1%) [33]. Half of the articles (7/14, 50%) reported the use of theoretical frameworks including sociocognitive theory [21,25,28], control theory [25,30], transtheoretical model [30,33], learning theory [23], operant conditioning, ecological theory, social network theory [25], cognitive behavioral therapy [32], and self-efficacy theory [28]. The attrition rate ranged from 4.8% [28] to 36.8% [21], and the follow-up time points ranged from 3 [21,28] to 24 months [25].

Intervention characteristics of the 14 included articles are detailed in Table 2. Most of the coaching sessions were delivered individually except 3 (21.4%) articles [26,27,33] that reported group health-coaching sessions. Most of the interventionists included dieticians (8/14, 57.1%) [20,22-24,26,30,31,33], psychologists (4/14, 28.6%) [23,26,27,32], endocrinologists (3/14, 24.1%) [20,23,29], health coaches (2/14, 14.3%) [25,28], physical activity coaches or physiologists (2/14, 14.3%) [27,31], nutritionists [21], physicians [33], pharmacists [33], and nurses [33]. Five (35.7%) articles mentioned some form of certification in nutrition, fitness, and lifestyle coaching [20,29,31-33]. Seven studies [20,21,24,26,31,32] reported control groups receiving apps, whereas 8 studies [22,23,25,27,29-31,33] reported control groups receiving usual care (without app or health coaching). Number of coaching sessions ranged from 2 [28] to 24 [23], and the program duration ranged from 2 [32,33] to 24 months [25]. Several behavioral change techniques have been used including instructions on how to perform behavior (ie, through educational materials and coaching; 14/14, 100%), self-monitoring of behaviors (ie, through automatic wearable devices or manual logging; 14/14, 100%), goal setting and planning (10/14, 71.4%) [20-23,26,28,30,32], prompts or cues (9/14, 64.3%) [20-24,26,29,30], social support (7/14, 50%), problem solving (6/14, 42.9%) [20-22,25,30], rewards (5/14, 35.7%) [22-24,30], and reduce negative emotions (ie, stress) (2/14, 14.3%) [23,30].

**Table 1:** Sample characteristics of the 14 included articles.

Author, year	Country	Sample size	Age (years), mean	BMI (kg/m^2^), mean	Follow-up (months)	Theoretical framework	Attrition (%)
Alencar et al [20], 2019	US	30	46.6	34.7	3	NS^a^	16.7
Allen et al [21], 2013	US	68	44.9	34.3	6	Social cognitive theory, motivational interviewing counseling techniques	36.8
Allman-Farinelli et al [30], 2016	Australia	250	27.6	26.9	3 and 9	Control theory, transtheoretical model	19.2
Bennett et al [22], 2018	US	351	50.7	35.9	6 and 12	NS	17.9
Block et al [23], 2015	US	339	55.0	31.2	3 and 6	Learning theory	13.9
Burke et al [24], 2021	US	502	45.0	33.7	6	NS	16.5
Godino et al [25], 2016	US	404	22.7	28.9	6, 12, 18, and 24	Social cognitive theory, control theory, and operant conditioning, ecological theory, social network theory	15.6
Hurkmans et al [31], 2018	Belgium	102	44.7	32.0	3	Behavioral change techniques such as self-monitoring, action planning, and relapse prevention	20.6
Kim et al [32], 2020	Korea	70	21.8	28.0	2 and 6	Cognitive behavioral therapy	20.0
Pagoto et al [26], 2021	US	64	39.8	34.2	3	NS	7.8
Spring et al [27], 2017	US	96	39.3	34.6	3, 6, and 12	NS	19.8
Stephens et al [28], 2017	US	62	20.0	28.5	3	Self-efficacy theory, construct of social cognitive theory	4.8
Tanaka et al [33], 2018	Japan	112	46.3	28.1	2 and 3	Transtheoretical model	27.7
Vaz et al [29], 2021	US	28	43.2	34.4	3 and 6	NS	14.3

^a^NS: not specified.

**Table 2:** Intervention characteristics of the 14 included articles.

Author, year	Group or individual	Interventionist/certification/automated or human-delivered	Control conditions	App name/external monitoring device	Coaching sessions, n	Duration (months)
Alencar et al [20], 2019	Individual	Endocrinologist, dietitians/level 2 certificate in weight management/human	Self-monitoring app	MyFitnessPal/accelerometer, blood pressure monitor, body composition scale (Withings)	12	3
Allen et al [21], 2013	Individual	Nutritionist/NS^a^/human	Self-monitoring app	Lose It!/NS	CG1^b^: 14; CG2: 7	6
Allman-Farinelli et al [30], 2016	Individual	Dietitian/NS/human	Text and static educational website	TXT2BFiT/NS	5	3
Bennett et al [22] 2018	Individual	Dietitian, student/NS/human	Usual care	Track/oscillometer device (Omron HEM 907XL)	18	12
Block et al [23], 2015	Individual	Diabetes educators, endocrinologists, dieticians, psychological experts/NS/automated	Waitlist control	Alive-PD/NS	36^c^	12^c^
Burke et al [24], 2021	Individual	Dietician/NS/human	Self-monitoring app	SMARTER/wireless scale and Fitbit Charge 2	20	6
Godino et al [25], 2016	Individual	Health coach/NS/human	Group education	GoalGetter app, BeHealthy app, TrendSetter app/calibrated digital scale (Seca 703, Seca GmbH & Co KG)	NS	24
Hurkmans et al [31], 2018^d^	Individual	Dietitian, physical activity coach/qualified physical activity coach/human	CG1: self-monitoring; CG2: waitlist control	NS/triaxial accelerometer (Acti Graph)	4	3
Kim et al [32], 2020	Individual	Psychologist/qualified behavioral therapist/human	Self-monitoring app	Noom Coach app/body composition analyser (InBody H20B Analyzer)	Unclear	2
Pagoto et al [26], 2021	Group	Dietitian, psychologist/NS/human	Self-monitoring app (MyFitnessPal)	Slip Buddy/Wi-Fi scale (Fitbit Aria)	12	3
Spring et al [27], 2017	Group	Psychologist, physiologist/NS/human	Group education with print resources	ENGAGED/accelerometer (TECH)	12	6
Stephens et al [28], 2017	Individual	Health coach/NS/human	Text and static educational website	Lose it!/body composition analyser (Tanita BS-549 scale)	2	3
Tanaka et al [33], 2018	Group	Dietitian, nurse, pharmacist, physician/certified by app company/human	Waitlist control	Wellness coach/calibrated digital scale (WB-150; Tanita)	NS	2
Vaz et al [29], 2021	Individual	Endocrinologist/certified by obesity medicine board/automated	Waitlist control	Fitbit app/Smart scale (Fitbit Aria)	NS	6

^a^NS: not specified.

^b^CG: control group.

^c^Alive-PD is supposed to be a 1-year program with weekly contacts for the first 6 months and biweekly thereafter, but in the included study, only the 6-month follow-up data were reported.

^d^Reported a 4-arm study comparing the effectiveness of a conventional face-to-face; app plus health coaching and app only weight loss program with a waitlist control group. As the conventional face-to-face program is not an intervention of interest, this study was analyzed as a 3-arm study. This was also the only study that compared the differences between app plus health coaching and app only weight loss program and a significant difference was reported. As 2 included articles reported 3-arm studies, the 14 articles were analyzed as 16 studies.

### Methodological Quality Appraisal

The overall ROB of each study was judged based on the highest ROB rating given for any of the 6 domains (Table S2 in Multimedia Appendix 2). Most of the studies were rated as having an unclear ROB (8/14, 57.1%) while the rest were judged to have a high ROB (6/14, 42.9%). Although all 14 studies were judged to have a low or unclear risk of selection bias due to randomization and allocation concealment, the highest ROB was due to the high attrition rate and an incomplete reporting of missing data management strategy (4/14, 28.6%). Concerning ROB was also found for performance (2/14, 14.3%) and detection bias (2/14, 14.3%). This suggests the need for future research to adhere to higher standards of methodological quality that improve the accuracy and usefulness of findings.

### Primary Outcomes

#### Overall Comparison

**Figure 2:**
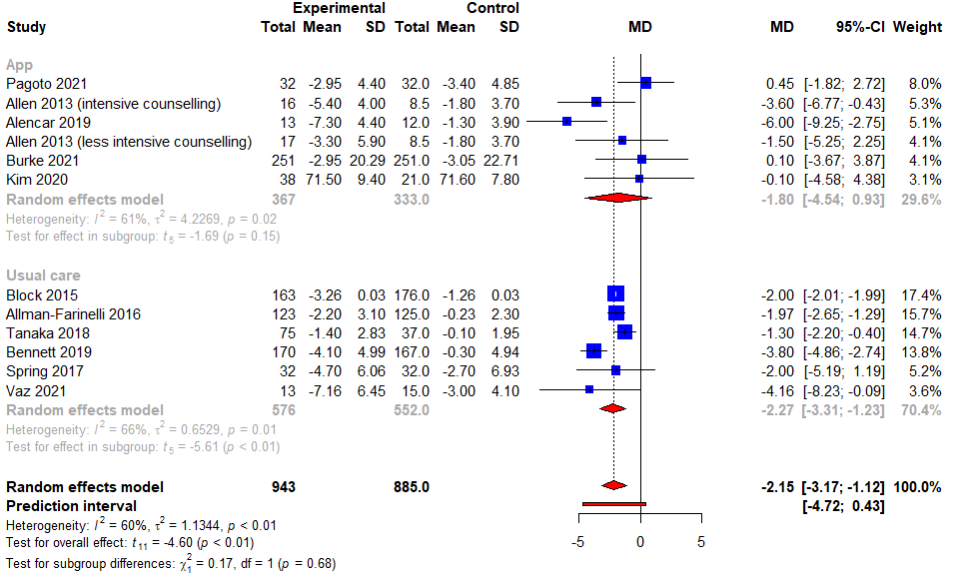
An illustration of the summary statistics of the intervention and control groups in each study included in the meta-analysis on the effect of smartphone self-monitoring apps with and without health coaching on weight (kg). The illustration also shows the subgroup analysis of the studies based on whether the control group received a smartphone self-monitoring app intervention or not. MD: mean difference.

**Figure 3:**
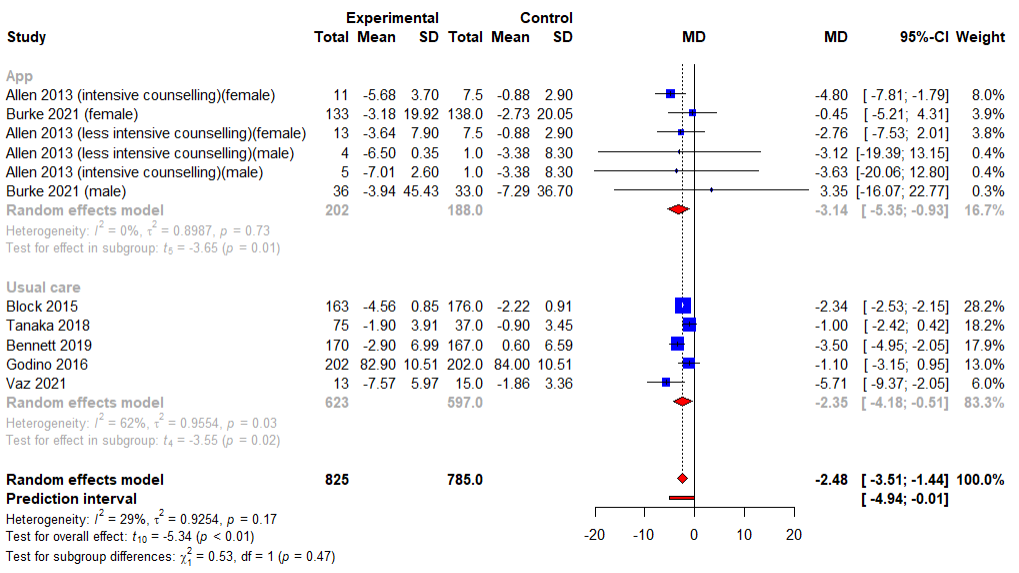
An illustration of the summary statistics of the intervention and control groups in each study included in the meta-analysis on the effect of smartphone self-monitoring apps with and without health coaching on waist circumference (cm). The illustration also shows the subgroup analysis of the studies based on whether the control group received a smartphone self-monitoring app intervention or not. MD: mean difference.

**Figure 4:**
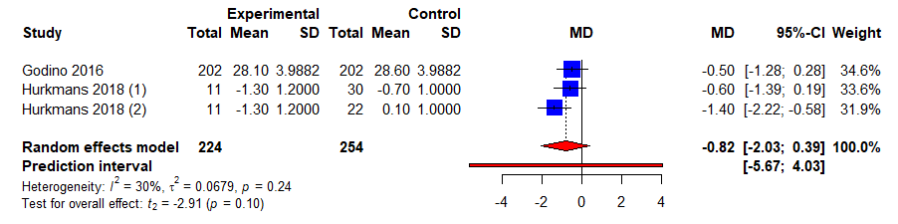
An illustration of the summary statistics of the intervention and control groups in each study included in the meta-analysis on the effect of smartphone self-monitoring apps with and without health coaching on body mass index (kg/m2). MD: mean difference.

A summary of the meta-analysis findings can be found in Table 3. Of 12 studies and 943 participants examined for weight loss in kg, combined intervention had significantly higher weight loss of 2.15 kg as compared to control arms (95% CI −3.17 kg to −1.12 kg; *P*<.001; *I*^2^=60.3%) (Figure 2 and Table 3). Of the 11 studies and 825 participants examined for change in WC, combined intervention led to a significant reduction of WC by 2.48 cm as compared to control arms (95% CI −3.51 cm to −1.44 cm; *P*<.001; *I*^2^=29.0%) (Figure 3 and Table 3). Of 3 studies and 224 participants examined for BMI, no significant differences were noted between combined intervention and control arms (WMD −0.82 kg/m^2^, 95% CI −2.03 kg/m^2^to 0.39 kg/m^2^; *P*=.1; *I*^2^=29.9%) (Figure 4 and Table 3). No publication bias for weight and WC was detected based on the visualization of funnel plot symmetry (−0.17; *t*=−0.34; *P*=.74) (Figure S1 in Multimedia Appendix 3 and Figure S2 in Multimedia Appendix 4) and Egger test (−0.11; *t*=−0.27; *P*=.79).

**Table 3:** A summary of the meta-analyses results of each outcome examined.

Outcomes	*k*	MD^a^ or SMD^b^ (95% CI)	*T* test	*P* value	τ^2^	*I*^*2*^ (%)
Weight (kg)	12	−2.15 (−3.17 to −1.12)	−4.60	<.001^c^	1.13	60.3
BMI (kg/m^2^)	3	−0.82 (−2.03 to 0.39)	−2.91	.10	0.07	29.9
Waist circumference (cm)	11	−2.48 (−3.51 to −1.44)	−5.34	<.001^c^	0.93	29.0
SBP^d^ (mm Hg)	6	−0.83 (−3.21 to 1.54)	−0.90	.41	<0.001	24.3
DBP^e^ (mm Hg)	6	−0.89 (−2.75 to 0.96)	−1.24	.27	<0.001	17.0
Body fat (%/kg)	3	−0.36^b^ (−1.90 to 1.18)	−1.00	.42	0.26	73.0
Total cholesterol (mg/dL)	2	2.82 (−2.86 to 8.51)	6.31	.10	0.00	0.0
LDL-C^f^ (mg/dL)	2	−3.78 (−10.05 to 2.49)	−7.66	.08	0.00	0.0
HDL-C^g^ (mg/dL)	2	1.63 (−16.00 to 19.25)	1.17	.45	1.79	42.6
Triglyceride (mg/dL)	4	−0.22 (−0.33 to 0.11)	−6.25	.008^h^	0.00	0.0
Glycated hemoglobin (%)	4	−0.12 (−0.21 to −0.02)	−3.8	.03^i^	0.002	35.7
Total calorie consumption/day (kcal)	5	−128.30 (−182.67 to −73.94)	−6.55	.003^h^	0.00	0.0
Physical activity	6	−0.11^b^ (−0.87 to 0.65)	−0.37	.72	0.36	72.8

^a^MD: mean difference.

^b^SMD: standardized mean difference adjusted with Hedges *g*.

^c^*P*<.001.

^d^SBP: systolic blood pressure.

^e^DBP: diastolic blood pressure.

^f^LDL-C: low density lipoprotein cholesterol.

^g^HDL-C: high density lipoprotein cholesterol.

^h^*P*<.01.

^i^*P*<.05.

#### Subgroup Analysis and Meta-Regression

A subgroup analysis was conducted based on the type of control conditions. For weight loss, subgroup analyses only showed a significant effect of the combined intervention when compared to usual care (WMD −2.27 kg, 95% CI −3.31 kg to −1.23 kg; *P*<.01; *I*^2^=66.0%) (Figure 2), but not with using apps (WMD −1.80 kg, 95% CI −4.54 kg to 0.93 kg; *P*=.15; *I*^2^=61.3%) (Figure 2). For reduction in WC, subgroup analysis revealed that combined intervention was superior to both using apps (*k*=5; WMD −2.35 cm, 95% CI −4.18 cm to −0.51 cm; *P*=.02; *I*^2^=62.0%), and usual care (*k*=6; WMD −3.14 cm, 95% CI −5.35 cm to −0.93 cm; *P*=.01; *I*^2^=0.0%).

A meta-regression on the number of coaching sessions showed no significant associations with weight (coefficient=0.002; *P*=.98; τ^2^_Unexplained_=2.29; *R*^2^=0.0) and WC (coefficient=0.104; *P*=.12; τ^2^_Unexplained_=0.84; *R*^2^=9.0).

### Secondary Outcomes: Overall Effect

**Figure 5:**
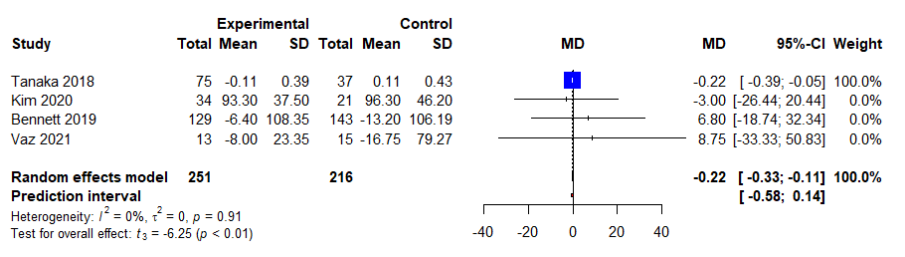
An illustration of the summary statistics of the intervention and control groups in each study included in the meta-analysis on the effect of smartphone self-monitoring apps with and without health coaching on triglyceride. MD: mean difference.

**Figure 6:**
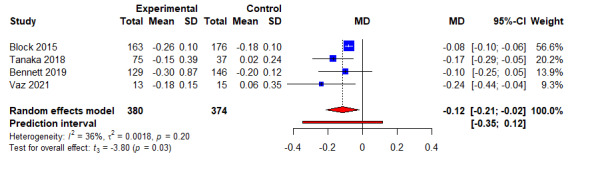
An illustration of the summary statistics of the intervention and control groups in each study included in the meta-analysis on the effect of smartphone self-monitoring apps with and without health coaching on hemoglobin A1c. MD: mean difference.

**Figure 7:**
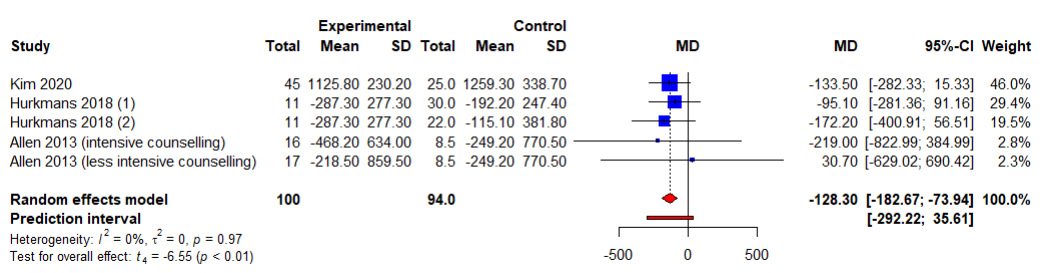
An illustration of the summary statistics of the intervention and control groups in each study included in the meta-analysis on the effect of smartphone self-monitoring apps with and without health coaching on total calorie consumption per day. MD: mean difference.

When compared to control arms, the combined intervention significantly reduced triglyceride (*k*=4; WMD −0.22 mg/dL, 95% CI 0.33 mg/dL to 0.11 mg/dL; *P*<.01; *I*^2^=0.0%) (Figure 5 and Table 3) and HbA_1c_ levels (*k*=4; WMD −0.12%, 95% CI −0.21 to 0.02; *P*=.03; *I^2^*=0.0%) (Figure 6 and Table 3). However, no significant differences were noted when combined intervention was compared to controls for other lipid parameters, such as TC (Figure S3 in Multimedia Appendix 5) [22,32], LDL-C (Figure S4 in Multimedia Appendix 6) [22,32], and HDL-C (Figure S5 in Multimedia Appendix 7) [22,33]. Next, combined intervention significantly reduced total calorie consumption per day as compared to controls (*k*=5; WMD −128.30 kcal, 95% CI −182.67 kcal to −73.94 kcal; *t*=−6.55; *P*=.003; *I*^2^=0.0%) (Figure 7 and Table 3), but no significant differences were noted for body fat percentage (Figure S6 in Multimedia Appendix 8 and Table 3) [21,24,32] and physical activity (Figure S7 in Multimedia Appendix 9 and Table 3). Finally, of the 6 included studies and 724 participants, no significant differences were noted between combined interventional and controls for both systolic blood pressure and diastolic blood pressure (Figure S8 in Multimedia Appendix 10, Figure S9 in Multimedia Appendix 11, and Table 3).

## Discussion

### Overview

A recent meta-analysis reported that health apps can lead to a significant weight loss of up to 2.18 kg but little is known about the effects of an adjuvant health coaching element [34]. Our study found that the use of mobile apps in conjunction with a patient-centered health coaching program significantly improves weight loss (2.15 kg), WC, triglyceride, HbA_1c_, and total calorie consumption per day as compared to usual care. However, the effect of an additional health coaching component to a self-monitoring app remains limited, as our subgroup analyses only found a significant improvement in WC, a surrogate for visceral fat, but not across the other cardiometabolic parameters. This suggests either a limited benefit of health coaching in addition to self-monitoring apps or the need for more rigorous health coaching components, such as more intensive feedback sessions, social support, and problem solving [35]. This is especially when most self-monitoring apps already encompass several behaviors change strategies, such as self-monitoring, goal setting, planning, and prompts or cues.

Behavioral modification that promotes healthy eating and physical activity is an essential and effective component of obesity management. However, most prior systematic reviews and meta-analyses had focused solely on clinical outcomes and not intermediary behavioral outcomes. Consistent with previous systematic reviews [36-38], our study found that smartphone apps are effective for weight loss and reducing HbA_1c_ levels. Additionally, our study adds to the current knowledge that smartphone apps with health coaching are effective means to improve eating behavior, specifically to reduce total calorie consumption. As behavioral change is a noninvasive and relatively low-cost intervention for obesity [39], using smartphone apps could be a scalable, sustainable, and effective approach to promoting healthy eating, especially with the increasing use of smartphone apps [40,41].

In contrast, we found no evidence of improvement in physical activity related to the use of smartphone apps with or without health coaching, potentially due to a small number of studies included for the analysis to be adequately powered. This finding is consistent with that from a pilot randomized controlled trial study conducted in England to evaluate the impact of a theory-based weight loss interventional program on behaviors [42]. The fact that weight management interventions had a positive impact on eating behavior but not on physical activity, this might indicate a need for different interventional approaches for different goals. Whereas smartphone apps that promote self-monitoring might be sufficient to motivate healthy eating, incorporating other mechanisms, such as financial or nonfinancial incentives (eg, rewards, praise for goal achievement, and reinforcement of positive behavioral changes) for weight loss could effectively increase physical activity [43,44].

Our study showed that combining smartphone apps with health coaching only yields a significantly greater reduction in WC but not weight loss as compared to using a self-monitoring app alone. This could be due to an increase in physical activity and hence muscles, resulting in a more toned body that reduces WC but not body weight. However, this conclusion was merely based on a subgroup analysis, which is prone to generating inaccurate results in this study false negatives, due to inadequate power [45]. Although one study reported improved outcomes when health coaching was used in addition to a smartphone app, the sample in each group was small, undermining the study’s reliability [46,47]. Interventional effects may also be influenced by personal intention, motivation [48], socioenvironmental resources, and interventional engagement for weight loss [49]. We also found intraprogram variations in health-coaching programs. The health coaching programs’ duration and context substantially varied, which may explain the nonsignificant effects we found for health coaching in conjunction with smartphone apps. Larger prospective studies examining a theory-based health coaching program are warranted to examine the effects of combining health coaching with smartphone apps for obesity-related outcomes.

### Strengths and Limitations

To our knowledge, this is the first meta-analysis conducted to summarize the attributable effects of health coaching in conjunction with smartphone self-monitoring apps, designed to improve obesity-related outcomes. We included health coaching based on the 5 patient-centric features (ie, goal setting, education, active learning, self-efficacy, and accountability) and examined whether providing health coaching provides additional benefits to smartphone apps. A key strength of this study is the use of the 5 criteria according to a well-established definition of health coaching to screen and include relevant articles, overcoming a notable limitation in the limited use of a clear definition of health coaching in the current literature [50]. This review has its limitations. First, the 16 included studies’ methodological quality varied substantially. However, we excluded low-quality studies to minimize the ROB. Second, researchers examined smartphone apps and health coaching with various design features, as described. To address this issue, we conducted meta-analyses using a random-effects model and accounted for interprogram heterogeneity, which indicated a small degree of heterogeneity. Third, our findings may not be generalizable to all populations across ages because the participants were young on average. Usability of and response to smartphone-based interventions may differ among older adults.

### Conclusions

This meta-analysis demonstrated that smartphone apps could be an effective means for certain obesity-related clinical and behavioral outcomes, such as weight loss, improvement in HbA_1c_ levels, and eating habits. We found no statistical evidence of additional benefits from using health coaching in conjunction with smartphone apps as compared to smartphone apps alone to improve obesity-related outcomes.

## Appendix

Multimedia Appendix 1 Table S1. Search strategy.

Multimedia Appendix 2 Table S2. Methodological quality assessment of 16 included articles using the Cochrane Risk of Bias tool.

Multimedia Appendix 3 Funnel plot of symmetry for the included studies that reported the effects of a smartphone self.

Multimedia Appendix 4 Funnel plot of symmetry for the included studies that reported the effects of a smartphone self.

Multimedia Appendix 5 An illustration of the summary statistics of the intervention and control groups in each study included.

Multimedia Appendix 6 An illustration of the summary statistics of the intervention and control groups in each study included.

Multimedia Appendix 7 An illustration of the summary statistics of the intervention and control groups in each study included.

Multimedia Appendix 8 An illustration of the summary statistics of the intervention and control groups in each study included.

Multimedia Appendix 9 An illustration of the summary statistics of the intervention and control groups in each study included.

Multimedia Appendix 10 An illustration of the summary statistics of the intervention and control groups in each study included.

Multimedia Appendix 11 An illustration of the summary statistics of the intervention and control groups in each study included.

## Abbreviations

HbA1c: glycated hemoglobin
HDL-C: high-density lipoprotein cholesterol
LDL-C: low-density lipoprotein cholesterol
mHealth: mobile health
NCCHWC: National Consortium for Credentialing Health and Wellness Coaches
PRISMA: Preferred Reporting Items for Systematic Reviews and Meta-Analysis
PROSPERO: International Prospective Register of Systematic Reviews
ROB: Risk of Bias
TC: total cholesterol
WC: waist circumference
WMD: weighted mean difference
