# Supplementary material for: Effectiveness of Combined Health Coaching and Self-Monitoring Apps on Weight-Related Outcomes in People With Overweight and Obesity: Systematic Review and Meta-analysis
Source: J Med Internet Res. 2023 Apr 18;25:e42432. doi: 10.2196/42432 (PMC10155083; doi:10.2196/42432)
Supplement: Multimedia Appendix 1 [file jmir_v25i1e42432_app1.docx]

# Table S1 Search strategy.

| Database | Index and keyword terms | Results |
| --- | --- | --- |
| *PubMed* | ((((App[Title/Abstract] OR application[Title/Abstract] OR mobile health[Title/Abstract] OR m-health[Title/Abstract] OR mhealth[Title/Abstract]) AND (counsel*[Title/Abstract] OR coach*[Title/Abstract] OR mentor* OR motivational interview*[Title/Abstract] OR positive psychology[Title/Abstract] OR facilitator*[Title/Abstract] OR instructor*[Title/Abstract] OR trainer*[Title/Abstract])) AND (Weight[Title/Abstract] OR bmi[Title/Abstract] OR body mass index[Title/Abstract])) AND (Obes*[Title/Abstract] OR overweight[Title/Abstract] OR high BMI[Title/Abstract] OR high body mass index[Title/Abstract])) AND (Randomized[Title/Abstract] OR randomised[Title/Abstract] OR randomly[Title/Abstract] OR random[Title/Abstract] OR control group[Title/Abstract]) | 115 |
| *EMBASE* | (app:ab,ti OR application:ab,ti OR 'mobile health':ab,ti OR 'm health':ab,ti OR mhealth:ab,ti) AND (counsel*:ab,ti OR coach*:ab,ti OR mentor*:ab,ti OR 'motivational interview*':ab,ti OR 'positive psychology':ab,ti OR facilitator*:ab,ti OR instructor*:ab,ti OR trainer*:ab,ti) AND (weight:ab,ti OR bmi:ab,ti OR 'body mass index':ab,ti) AND (obes*:ab,ti OR overweight:ab,ti OR 'high bmi':ab,ti OR 'high body mass index':ab,ti) AND (randomized:ab,ti OR randomised:ab,ti OR randomly:ab,ti OR random:ab,ti OR 'control group':ab,ti) | 119 |
| *The Cochrane Library (trials only)* | App OR application OR mobile health OR m-health OR mhealth in Title Abstract Keyword AND counsel* OR coach* OR mentor* OR motivational interview* OR positive psychology OR facilitator* OR instructor* OR trainer* in Title Abstract Keyword AND Weight OR bmi OR body mass indexment in Title Abstract Keyword AND Randomized OR randomised OR randomly OR random OR control group in Title Abstract Keyword AND Obes* OR overweight OR high BMI OR high body mass index in Title Abstract Keyword - (Word variations have been searched) | 456 |
| *CINAHL* | AB ( App OR application OR mobile health OR m-health OR mhealth ) AND AB ( counsel* OR coach* OR mentor* OR motivational interview* OR positive psychology OR facilitator* OR instructor* OR trainer* ) AND AB ( Weight OR bmi OR body mass index ) AND AB ( Obes* OR overweight OR high BMI OR high body mass index ) AND AB ( Randomized OR randomised OR randomly OR random OR control group ) | 61 |
| *PsycInfo* | ((App or application or mobile health or m-health or mhealth) and (counsel* or coach* or mentor* or motivational interview* or positive psychology or facilitator* or instructor* or trainer*)).af. and (Weight or bmi or body mass index).ab. and (Obes* or overweight or high BMI or high body mass index).ab. and (Randomized or randomised or randomly or random or control group).ab. | 169 |
| *Scopus* | ( TITLE-ABS-KEY ( app OR application OR "mobile health" OR m-health OR mhealth ) AND ALL ( counsel* OR coach* OR mentor* OR "motivational interview*" OR positive AND psychology OR facilitator* OR instructor* OR trainer* ) AND TITLE-ABS-KEY ( weight OR bmi OR "body mass index" ) AND TITLE-ABS-KEY ( obes* OR overweight OR "high BMI" OR "high body mass index" ) AND TITLE-ABS-KEY ( randomized OR randomised OR randomly OR random OR "control group" ) ) | 284 |
| *Web of Science* | App OR application OR mobile health OR m-health OR mhealth (Topic) and counsel* OR coach* OR mentor* OR motivational interview* OR positive psychology OR facilitator* OR instructor* OR trainer* (Topic) and Weight OR bmi OR body mass index (Topic) and Obes* OR overweight OR high BMI OR high body mass index (Topic) and Randomized OR randomised OR randomly OR random OR control group (Topic) | 218 |
