# Supplementary material for: Effectiveness of Combined Health Coaching and Self-Monitoring Apps on Weight-Related Outcomes in People With Overweight and Obesity: Systematic Review and Meta-analysis
Source: J Med Internet Res. 2023 Apr 18;25:e42432. doi: 10.2196/42432 (PMC10155083; doi:10.2196/42432)
Supplement: Multimedia Appendix 2 [file jmir_v25i1e42432_app2.docx]

# Table S2 Methodological quality assessment of 16 included articles using the Cochrane Risk of Bias tool.

| **Study** | **Random sequence generation** | **Allocation concealment** | **Blinding of participants and personnel** | **Blinding of outcome assessment** | **Incomplete outcome data** | **Selective reporting** | **Overall** |
| --- | --- | --- | --- | --- | --- | --- | --- |
| Alencar, 2019 | Low | Unclear | Low | Low | Low | Low | Unclear |
| Allen, 2013 | Low | Unclear | Unclear | Unclear | High | Low | High |
| Allman-Farinelli, 2016 | Low | Low | Low | Unclear | Low | Low | Unclear |
| Bennet, 2018 | Low | Unclear | Low | Unclear | Low | Low | Unclear |
| Block, 2015 | Low | High | Low | Unclear | Low | Low | High |
| Burke, 2021 | Low | Unclear | Unclear | Unclear | Low | Low | Unclear |
| Godino, 2016 | Low | Unclear | High | Low | Low | Low | High |
| Hurkmans, 2018 | Low | Unclear | Low | Low | High | Low | High |
| Kim, 2020 | Low | Unclear | High | High | High | Low | High |
| Pagoto, 2021 | Low | Unclear | Unclear | Unclear | Unclear | Low | Unclear |
| Spring, 2017 | Low | Unclear | Unclear | Low | Low | Low | Unclear |
| Stephens, 2017 | Low | Unclear | Unclear | Unclear | Unclear | Unclear | Unclear |
| Tanaka, 2018 | Low | Low | Low | Low | High | Low | High |
| Vaz, 2021 | Low | Unclear | Unclear | High | Unclear | Low | Unclear |
