# Supplementary material for: Effectiveness of Combined Health Coaching and Self-Monitoring Apps on Weight-Related Outcomes in People With Overweight and Obesity: Systematic Review and Meta-analysis
Source: J Med Internet Res. 2023 Apr 18;25:e42432. doi: 10.2196/42432 (PMC10155083; doi:10.2196/42432)

# Figure S2 Funnel plot of symmetry for the included studies that reported the effects of a smartphone self-monitoring with health coaching app on waist circumference.
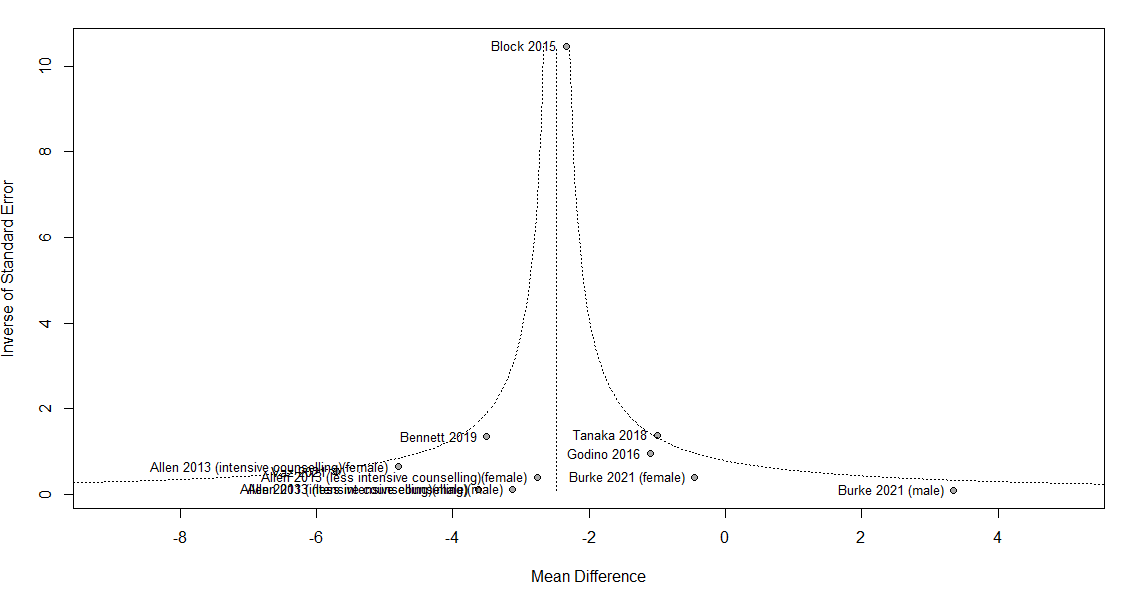

Supplement: Multimedia Appendix 4 [file jmir_v25i1e42432_app4.docx]
