# Supplementary material for: Effectiveness of Combined Health Coaching and Self-Monitoring Apps on Weight-Related Outcomes in People With Overweight and Obesity: Systematic Review and Meta-analysis
Source: J Med Internet Res. 2023 Apr 18;25:e42432. doi: 10.2196/42432 (PMC10155083; doi:10.2196/42432)

# Figure S4 An illustration of the summary statistics of the intervention and control groups in each study included in the meta-analysis on the effect of smartphone self-monitoring apps with and without health coaching on low-density lipoprotein cholesterol.
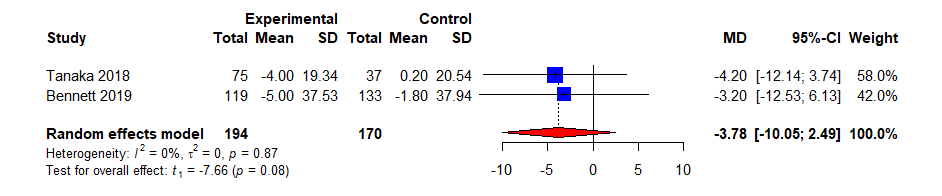

Supplement: Multimedia Appendix 6 [file jmir_v25i1e42432_app6.docx]
